# A Hybrid III stepped wedge cluster randomized trial testing an implementation strategy to facilitate the use of an evidence-based practice in VA Homeless Primary Care Treatment Programs

**DOI:** 10.1186/s13012-017-0563-2

**Published:** 2017-04-04

**Authors:** Molly M. Simmons, Sonya Gabrielian, Thomas Byrne, Megan B. McCullough, Jeffery L. Smith, Thom J. Taylor, Tom P. O’Toole, Vincent Kane, Vera Yakovchenko, D. Keith McInnes, David A. Smelson

**Affiliations:** 1 VA National Center on Homelessness among Veterans, Washington, DC USA; 2 VA Center for Health Organization and Implementation Research, Bedford, MA USA; 3 Boston University School of Public Health, Boston, MA USA; 4 VA Greater Los Angeles Health Care System, Los Angeles, CA USA; 5 UCLA David Geffen School of Medicine, Los Angeles, CA USA; 6 Boston University School of Social Work, Boston, MA USA; 7 College of Medicine, University of Arkansas for Medical Sciences, Little Rock, AR USA; 8 VA Palo Alto Health Care System, Palo Alto, CA USA; 9 VA Quality Enhancement Research Initiative for Team-Based Behavioral Health, Palo Alto, CA USA; 10 Alpert Medical School at Brown University, Providence, RI USA; 11 VA Medical Center Wilmington, Wilmington, DE USA; 12 VA Bridging the Care Continuum-Quality Enhancement Research Initiative, Bedford, MA USA; 13 University of Massachusetts Medical School, Worcester, MA USA

**Keywords:** Facilitation, Step wedge design, Co-occurring disorders, Vulnerable populations

## Abstract

**Background:**

Homeless veterans often have multiple health care and psychosocial needs, including assistance with access to housing and health care, as well as support for ongoing treatment engagement. The Department of Veterans Affairs (VA) developed specialized Homeless Patient Alignment Care Teams (HPACT) with the goal of offering an integrated, “one-stop program” to address housing and health care needs of homeless veterans. However, while 70% of HPACT’s veteran enrollees have co-occurring mental health and substance use disorders, HPACT does not have a uniform, embedded treatment protocol for this subpopulation. One wraparound intervention designed to address the needs of homeless veterans with co-occurring mental health and substance use disorders which is suitable to be integrated into HPACT clinic sites is the evidence-based practice called Maintaining Independence and Sobriety through Systems Integration, Outreach, and Networking-Veterans Edition, or MISSION-Vet. Despite the promise of MISSION-Vet within HPACT clinics, implementation of an evidence-based intervention within a busy program like HPACT can be difficult. The current study is being undertaken to identify an appropriate implementation strategy for MISSION-Vet within HPACT. The study will test the implementation platform called Facilitation and compared to implementation as usual (IU). The aims of this study are as follows: (1) Compare the extent to which IU or Facilitation strategies achieve fidelity to the MISSION-Vet intervention as delivered by HPACT homeless provider staff. (2) Compare the effects of Facilitation and IU strategies on the National HPACT Performance Measures. (3) Compare the effects of IU and Facilitation on the permanent housing status. (4) Identify and describe key stakeholders’ (patients, providers, staff) experiences with, and perspectives on, the barriers to, and facilitators of implementing MISSION.

**Design:**

Type III Hybrid modified stepped wedge implementation comparing IU to Facilitation across seven HPACT teams in three sites in the greater Los Angeles VA system. This is a cluster randomized trial.

**Discussion:**

Integrating MISSION-Vet within HPACT has the potential to improve the health of thousands of veterans, but it is crucial to implement the intervention appropriately in order for it to succeed. The lessons learned in this protocol could assist with a larger roll-out of MISSION within HPACT. This protocol is registered with clinicaltrials.gov and was assigned the number NCT 02942979.

## Background

Veterans comprise 10% of the homeless population in the USA and often have multiple complex health care and psychosocial needs that threaten housing stability [1, 2]. To address these needs, the Department of Veterans Affairs (VA) developed specialized Homeless Patient Aligned Care Teams (HPACT). The goal of HPACT is to offer an integrated “one-stop program” that takes into account social determinants of health to address medical and mental health, substance use disorder (SUD), and psychosocial needs (e.g., housing, benefits) of homeless veterans [3]. This is consistent with the patient-centered medical home model that is commonly delivered within and outside of the VA system [4, 5] but different in that HPACT is specifically tailored to address complex needs of homeless veterans.

While HPACT have a strong track record of improving care for homeless veterans [1], the main focus is engagement in primary care and enhancing access to specialty care for this vulnerable population. However, though most (70%) of HPACT’s enrollees have co-occurring mental health and substance use disorders, it does not have a uniform, embedded treatment protocol for these patients and often addresses these disorders through referrals to specialty care. Co-occurring mental health and substance use disorders are a risk factor for homelessness and can result in higher rates of treatment disengagement, poor community integration and higher utilization of costly emergency department, and inpatient mental health and medical services [1].

One wraparound intervention designed to address the needs of homeless veterans with a co-occurring mental health and substance use disorders which is suitable for integration into HPACT clinic sites is the evidence-based practice called Maintaining Independence and Sobriety through Systems Integration, Outreach, and Networking-Veterans Edition, or MISSION-Vet. Studies to date suggest that MISSION-Vet, when utilized with homeless individuals, improves treatment engagement for individuals with co-occurring mental health and substance use disorders and with mental health and substance abuse outcomes and reduces the number of days homeless and re-hospitalizations [6, 7]. Based on literature supporting the efficacy of MISSION-Vet, it has recently been added to the Substance Abuse and Mental Health Services Registry of Evidence-Based Practices [8–11]. Implementation of MISSION-Vet within HPACT would fill the current gap by integrating treatment of co-occurring mental health and substance use disorders within HPACT clinics as opposed to addressing the needs through referrals which can fragment care and increase engagement issues.

Despite the promise of MISSION-Vet within HPACT clinics, implementation of an evidence-based intervention within a busy program like HPACT can be difficult [12–15]. It is known that in settings like HPACT clinics, site training and offering treatment manuals through the web alone, while less burdensome and resource intensive, are insufficient [12–15]. Therefore, the current study aims to identify an appropriate implementation strategy for MISSION-Vet within HPACT in the Greater Los Angeles (GLA) VA system to address the needs of homeless veterans with co-occurring mental health and substance use disorders. The project will be comparing implementation as usual (IU) to Facilitation at the facility level. Facilitation offers a process by which site level staff are assisted and supported in implementing and sustaining a new program or practice [16]. This paper describes the implementation protocol for this study.

## Methods

### Overview

The hypothesis of this project is that Facilitation will yield better adoption and fidelity to MISSION-Vet among HPACT clinics compared to IU. To test this hypothesis, we will utilize a type III Hybrid modified stepped wedge implementation design [17], meaning this is a cluster study design. Type III stepped wedge designs are increasingly being used in health services research because they offer a way to assess both the implementation strategy in the facility level and health outcomes [18, 19]. This project will implement MISSION-Vet within GLA’s HPACT teams across three facilities (West LA VA Medical Center, Sepulveda, and Downtown). In keeping with our study design, instead of starting all intervention and control sites together, the design will stagger introduction of Facilitation.

### HPACT

The HPACT model is based around four key elements: (1) open access, meaning care on-demand which is not reliant on mail/telephone-based scheduling and limited appointments; (2) homeless-tailored care; (3) intensive case management/high-frequency care meaning an ambulatory-intensive care unit (ICU) approach with high-frequency care; and (4) staff trained to work with homeless veterans with attention to engagement of this population [20]. Consistent with other medical home models, within HPACT, all of the medical services required by the veteran are typically co-located [20].

There are 60 HPACT clinics nationally. Currently, HPACT serve almost 18,500 veterans, 84.86% of whom receive mental health care, which includes substance abuse services. HPACT has proven successful: internal VA data show that among those enrolled in HPACT, there is a 25% reduction in emergency department (ED) visits, 25% reduction in hospitalizations, and more consistent care where patients average four primary care visits per year.

In GLA VA specifically, local VA data examining medical complexity scores of their HPACT clients suggests that those served are often sicker than the average HPACT patient nationally. For this reason, engagement can be problematic, particularly for homeless veterans with co-occurring mental health and substance use disorders. For example, in the last 7 months, the 4018 GLA HPACT veterans averaged 0.28 visits per person per month for those referred to substance use care compared to the program’s national average of 0.51. In the last 4 months, inpatient hospitalizations for GLA have increased 30% after HPACT enrollment. In contrast, HPACT has nationally reduced inpatient admissions by 7%. Given the complexity of GLA HPACT patients, a comprehensive intervention like MISSION-Vet could help the GLA HPACT clients get both access to and engaged in treatment services for co-occurring mental health and substance use disorders.

### MISSION-Vet clinical intervention

In MISSION-Vet, services are provided by a team comprised of a case manager and a peer team. Each veteran should, with optimal fidelity, receive 2.5 h of team support weekly. A core component of MISSION-Vet is ritical Time Intervention which includes both assertive outreach and structured psychoeducation sessions [21], a time-limited assertive case management model. Critical Time Intervention is intended to reduce the risk of homelessness through additional support to individuals with mental illness during the transition from institutions (e.g., inpatient psychiatry units, residential treatment programs, and homeless shelters) to community living or to engage individuals already living in the community in care. These Critical Time Intervention services are delivered in three phases of decreasing intensity: Transition to Community, Try-Out, and Transfer of Care. During the Transition to Community phase, services are intended to reinforce community living and linkages to key community support services to build a safety net within the community. During the Try-Out phase, the case manager/peer specialist team begins to readjust the community-based support systems to fill any gaps and reduce service intensity to help the veteran test what it is like to live in the community. Finally, visits during the Transfer of Care phase are used to fine-tune connections with community-based resources.

Additionally, MISSION-Vet supplements Critical Time Intervention with 13 dual recovery therapy (DRT) psychoeducational sessions which are delivered by the case manager [22–24]. These sessions, which are highly structured, include the use of motivational interviewing [25] and relapse prevention [26] techniques. They specifically target issues commonly facing homeless veterans with co-occurring mental health and substance use disorders. MISSION-Vet also includes 11 structured psychoeducational sessions with peer specialists that are designed to empower veterans to plan for a life of stability, sobriety, and community integration. In addition to Critical Time Intervention, DRT, and Peer Support, MISSION-Vet case managers/peer specialists also offer assistance with employment and are trained to deliver trauma-informed services, which have been found to be effective with our target population [27, 28]. While each component of MISSION-Vet has demonstrated efficacy on its own, when combined using the MISSION-Vet platform, they work together to increase treatment engagement, improve mental health and substance abuse outcomes, and reduce ER visits, re-hospitalizations, and recurring homelessness [6, 7, 29].

Delivery of MISSION-Vet is guided by a Treatment Manual [30], which serves as a how-to-guide that describes MISSION-Vet’s core components and suggestions for service delivery and includes a number of appendices with additional didactic materials. Peer specialists also give veterans the MISSION-Vet Consumer Workbook [31] at the onset of treatment. This is intended to promote treatment engagement. This workbook is designed to serve as a complement to the Treatment Manual and help veterans integrate DRT and peer support concepts and increase their engagement in outpatient services through homework assignments, readings, and checklists.

In addition to the MISSION-Vet Treatment Manual and Consumer Workbook, MISSION-Vet also includes a structured webinar training. These trainings last 2 h and provide an overview of MISSION-Vet’s treatment model. They also offer an introduction to developed materials (manuals and fidelity measure). The training also covers the role of the supervisor, whose job is to provide ongoing consultation ensuring fidelity to MISSION-Vet.

### Passive implementation: implementation as usual

Passive implementation or IU for MISSION-Vet is comprised of the aforementioned 2 h webinar training, along with key information on how to access and use the MISSION-Vet Treatment Manual and Consumer Workbook. The manual is posted on the web and available inside the VA on the National Center for Homelessness among Veterans website or at missionmode.org. This passive strategy has been used in previous studies [32].

### Implementation platform: Facilitation

Facilitation is a comprehensive approach in which implementation experts partner with local staff to support implementation planning and to tailor adoption strategies to local contexts [33, 34]. Facilitation is rooted in the belief that while evidence to support a practice is important, it is equally important to draw on local clinical experience and professional knowledge to tailor implementation to local priorities, preferences, and resources [35]. Thus, Facilitation gives attention to addressing individual- and organizational-level factors that can influence successful implementation of an evidence-based practice with good fidelity [16, 20, 35].

To accomplish this, implementation experts serving as facilitators work to establish supportive relationships with those at the participating clinical setting to focus on implementation planning, goal-setting, and problem-solving [36]. In this study, Facilitation includes external and internal facilitators working in tandem to support MISSION-Vet implementation.

External facilitators (EF) are external to the implementation site and have general expertise in implementation strategies and quality improvement processes. Internal facilitators (IF) are located at the implementation site, meaning they are familiar with facility-level organizational structures, procedures, culture, and clinical policy and processes within the system at regional or local facility level. The EF and IF work collaboratively to present sites with design considerations and steps to implement evidence-based practices and offer ongoing data monitoring to identify gaps and solutions for implementation. The EF works during the course of the project to transfer knowledge and understanding of effective implementation strategies and processes to the IF, thus fostering introduction and retention of skills into the local organization so they can be leveraged not only for the current effort, but also for future quality improvement activities.

**Fig. 1:**
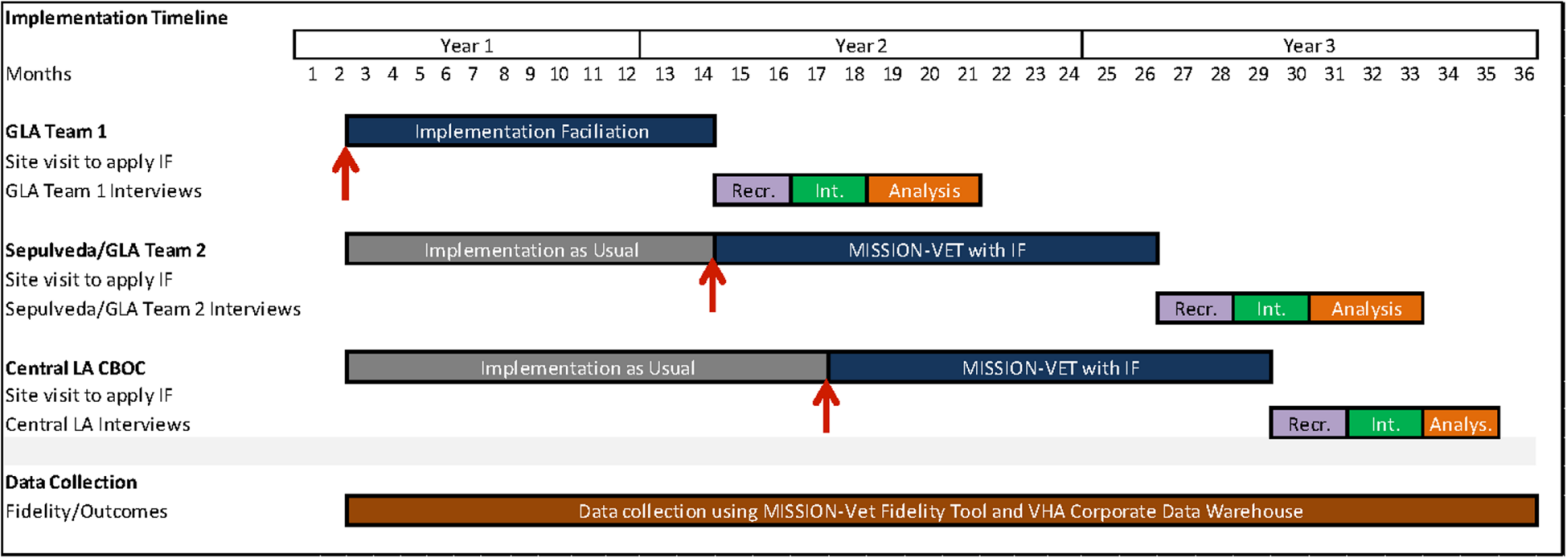
Project timeline

As depicted in Fig. 1, for this project, Facilitation will be initiated in month 7 of year 1 and run for 12 months at each site. In operationalizing Facilitation, a research team member (JLS) with extensive experience and expertise in applying external facilitation strategies to support adoption of evidence-based practices will serve as EF [2, 19, 37, 38]. A clinical team member from the GLA VA will serve as IF. During the first 6 months of the project, the IF will receive general training on Facilitation and on the role of the IF. This training will be conducted in GLA by the EF. Once trained, the IF, mentored and supported by the EF, will partner directly with GLA VA HPACT programs to guide implementation of MISSION-Vet as prescribed in the IF model—more specifically, by helping HPACT staff to develop local plans, goals, and objectives for MISSION-Vet implementation; to secure needed resources for successful implementation; and to ensure all aspects of MISSION-Vet are well planned, including addressing organizational barriers and tailoring to local conditions, monitoring implementation progress and fidelity, problem-solving to address barriers, and working to ensure MISSION-Vet sustainability.

### Participation sites and recruitment

#### Site composition

This study includes seven HPACT teams across three sites at the GLA VA. We selected GLA VA because of the size and scope of the homeless population: LA County has the second highest homeless population nationally, and GLA serves more homeless veterans than any VA in the nation. Within each HPACT team, there are two groups of subjects: one is case managers and peers and the other is HPACT veterans. Each team is comprised of staff at an HPACT clinic. The teams are composed of the following: teams A–D (West LA VA Medical Center; 44 HPACT team members and staff and 3100 veterans in total), team E (Sepulveda; 7 HPACT team members and staff and 407 veterans), and team F (Downtown; 8 HPACT team members and staff and 528 veterans), for a total of 59 HPACT team members and staff and 4018 veterans.

### Recruitment

All staff will be recruited into training at the three GLA sites. Consent will be obtained prior to training. Training will also include information about how the HPACT staff (in IU and Facilitation groups) can identify eligible veterans who meet the MISSION-Vet program criteria and begin to engage them in MISSION-Vet services. Engaging veterans into MISSION-Vet will take place on a rolling basis as the intervention is activated at each site. While this study targets case managers for recruitment, we will encourage case managers to follow the same recommended inclusion and exclusion criteria that are appropriate for their client’s participation in MISSION-Vet: (1) meet the *Diagnostic and Statistical Manual of Mental Disorders*, 5th Edition [39], diagnostic criteria of International Classification of Diseases, 10th Revision [40], for a current substance abuse or dependence disorder (e.g., alcohol, marijuana, cocaine) or polysubstance use and a co-occurring mental illness that includes anxiety, mood, or a psychotic spectrum disorder, (2) is willing to participate in the service, and (3) is empaneled in HPACT at one of the study sites.

### Study design

This study will use a stepped wedge design, which is a form of a cluster randomized study in which the intervention is sequentially implemented in all clusters of over a number of set time periods. Order of the start time of the intervention in each cluster is determined by random assignment. In a stepped wedge design, there is an initial time period in which no clusters receive the intervention, and all clusters cross over into the intervention condition by the end of the study. In the present study, the aforementioned HPACT clinics will form the naturally occurring clusters and Facilitation will be implemented in each clinic, with the start order for each clinic being randomly assigned by a random number generator. This allows us to evaluate the impact of the intervention but does not deprive any site eventually receiving the intervention [41]. This design will also allow us to identify and control for secular trends better than parallel groups randomized controlled trials [41].

The intervention will be implemented in two of four teams in the first site (site A) in the first step of the wedge. One will receive IU and the other Facilitation. After the initial phase of Facilitation, implementation will be applied in the other two GLA medical center teams and the other sites (site B and C) in a stepwise fashion as described below. Figure 1 includes a specific timeline for the study design.

This design will allow all sites to receive 1 year of Facilitation, and all sites but the first two teams (the pilot sites) to have a 1 year IU observation period and serve as its own control.

### Measures, procedures, and analyses by study aim

#### Aim 1: Compare the extent to which Facilitation and implementation as usual (IU) strategies achieve fidelity to the MISSION-Vet intervention as delivered by HPACT homeless provider staff

##### Data sources

MISSION-Vet Fidelity Measure; Evidence-Based Practice Attitudes scale; Organizational Support for Evidence-Based Practices scale [42–45]

##### Measures and data collection

The Clinical Note Template embedded within the VA’s computerized patient record system will be used at all sites to document the degree to which Facilitation enhances fidelity over and above IU. The MISSION-Vet Fidelity Measure tracks all the core elements of the MISSION-Vet treatment model, including Critical Time Intervention, DRT, Peer Support, vocational supports, and trauma-informed care for each individual veteran. The fidelity index consists of 78 items assessing the presence or absence of certain activities within MISSION-Vet and will be captured in veterans’ electronic medical records. Taking the responses from all non-missing items, we will compute a fidelity score for each veteran. Data on all HPACT veterans at all sites will be obtained from electronic medical records available in the VA’s Corporate Data Warehouse. The Evidence-Based Practice Attitudes scale and the Organizational Support for Evidence-Based Practices scale [42–45] will provide information on potential mediating factors. We will administer this measure to all HPACT staff annually throughout the study period.

##### Hypothesis

Fidelity will be superior in Facilitation compared to IU.

##### Data analysis

A mixed-effect linear regression model will be used to compare Facilitation and IU conditions on the fidelity tool’s total score after 1 year of Facilitation, with a random intercept included in the model to account for clustering of veterans within HPACT team members. Assuming an effect is observed for Facilitation vs. IU, we will further explore potential moderators to this effect with two-way interaction terms between Facilitation and potential moderating factors.

#### Aim 2: Compare the effects of Facilitation and IU strategies on the National HPACT Performance Measures

##### Data sources

We will rely on data from the Corporate Data Warehouse that captures inpatient and outpatient services utilization to assess the effect of Facilitation (relative to IU) on (1) inpatient hospitalizations and (2) engagement in substance abuse services.

##### Measures and data collection

We will use Corporate Data Warehouse data to construct measures of inpatient hospitalizations and engagement in substance abuse services. We will measure the number of medical, mental health, and overall inpatient hospitalization days in each month of the study’s observation period. Engagement in substance abuse services will be measured by the number of outpatient visits in a VA substance abuse specialty clinic, which will be identified based on clinic stop codes, in each month of the study’s observation period.

##### Hypothesis

Veterans will show greater improvement as indicated by reduced inpatient hospitalization days and increased engagement in substance abuse services (i.e., a greater number of visits in substance abuse specialty clinics) when receiving Facilitation compared to when receiving IU.

##### Data analysis

We will assess the effect of Facilitation using the modeling approach for stepped wedge designs outlined by Hussey and colleagues [41]. Specifically, this will entail modeling the outcomes described above using a three-level mixed-effect regression model in which the covariate of interest is a fixed effect for Facilitation (vs. IU), and that includes fixed effects for each time period to control for temporal trend, as well as random effects to account for clustering within veterans over time, HPACT case managers, and HPACT teams. As these outcomes are not likely to be normally distributed, we will explore several models (e.g., linear mixed models in which the outcome measure is log transformed, Poisson or negative binomial mixed models) to determine the best approach for modeling these outcomes.

#### Aim 3: Compare the effects of IU and Facilitation on permanent housing status

##### Data sources

Data on permanent housing placement and utilization of VA specialized homeless programs obtained from the VA National Homeless Registry.

##### Measures and data collection

We will create separate measures of (1) the number of days spent in VA specialized homeless programs (e.g., Grant and Per Diem, Domiciliary Care for Homeless Veterans, Supportive Services for Veteran Families rapid re-housing) for which veterans meet the statutory definition of homelessness; (2) number of days in VA permanent housing programs for which veteran is considered housed; and (3) number of days residing in the community (i.e., veteran is in neither VA homeless or VA permanent housing programs) during each month of the study period.

##### Hypothesis

Veterans will spend more days in VA permanent housing programs and in the community, and fewer days in VA specialized homeless programs when receiving Facilitation compared to IU.

##### Data analysis

We will use an analytic approach that parallels that described in Aim 2. Specifically, we will assess the impact of IU by using mixed-effects regression models in which the covariate of interest is a fixed effect for Facilitation (vs. IU), and that includes fixed effects for each time period to control for temporal trend, as well as random effects to account for clustering within veterans over time, HPACT case managers, and HPACT teams.

#### Aim 4: Identify and describe key stakeholders’ (patients, providers, staff) experiences with, and perspectives on, the barriers to and facilitators of implementing MISSION

##### Data sources

Semi-structured interviews will be conducted for a formative (FE) and summative evaluation (SE). The FE interviews will elicit key stakeholders’ experiences with, and perspectives on MISSION-Vet training, barriers to and facilitators of implementing MISSION-Vet as well as to identify particular areas to target for Facilitation. Summative evaluation interviews will identify stakeholder’s experiences with Facilitation and needed adaptations to MISSION-Vet as well as veteran experiences with MISSION-Vet.

##### Measures and data collection

All semi-structured interviews will be conducted by phone. For the FE, 13–18 individual, semi-structured qualitative interviews will be conducted with HPACT staff trained in MISSION-Vet. For the SE, participants will include 12 patients (two per site) and 15–20 providers/staff. Interview questions will focus on evaluating the implementation of Facilitation, IU, and MISSION-Vet with particular focus on barriers to Facilitation, perceptions of strengths and weaknesses of MISSION-Vet for this population and setting, and leadership support for implementation in HPACT.

The goal of the qualitative portion of this project is to get an understanding of what staff thought about MISSION-Vet itself and for researchers to understand the organizational context and then to identify barriers and facilitators to implementation of MISSION-Vet so that these areas can be addressed through Facilitation once it is activated.

##### Data analysis

Each interview will be recorded and transcribed verbatim. A codebook will be developed with a priori codes based on Consolidated Framework for Implementation Research [46] along with emergent thematic coding [47]. For the FE, interviews will be coded and analyzed to synthesize data on organizational strengths and weaknesses and to identify key areas for the Facilitation strategy to target. The same process will be followed with the SE. The SE will enable us to identify the differences and similarities experienced by participants both within LA sites and across the sites. These similarities and differences within and across sites will serve as a key “lessons learned” for subsequent efforts to facilitate use of MISSION-Vet across a larger number of sites in the VA.

### Power analysis

We conducted power calculations for Aims 1, 2, and 3 with the aid of the pwr package [48] in the *R* environment for statistical computing version 3.2.4 [49] and found that the anticipated sample sizes for all three aims have sufficient statistical power by conventional guidelines (i.e., 80% power to detect intervention effects at the 0.05 significance level). Aim 4 is a qualitative aim and we therefore did not conduct power calculations for that aim. Specific details on the power calculations for each of the quantitative aims are provided below and are based on an expected sample size of 1808 veterans and 53 HPACT staff members. This sample size estimate was based on the known rates of mental health and SUD disorders among HPACT participants, which suggest that conservatively, half of the 4018 veteran HPACT participants (or 2009 veterans) will meet the eligibility criteria for MISSION-Vet and participate in the study. The estimates also account for an anticipated 10% refusal among the 59 GLA HPACT staff members, which is assumed to result in an additional 10% decrease in the number of veteran participants.

Aim 1 involves an analysis of a cross-sectional outcome, and we thus calculated statistical power by correcting for the clustering of veterans within HPACT staff members, but not for the stepped wedge design. To do so, we calculated a “design effect” (Deff), which is commonly used in cluster randomized studies to estimate the effective sample size and resulting statistical power after accounting for clustering. We calculated the design effect using the formula Deff = 1 + *(n* − 1) × *ρ*, where *n* is the number of subjects within a cluster (in this case number of veterans per HPACT staff member) and *ρ* is the intraclass correlation coefficient (ICC) [50]. The value of *n* is 1808/53 = 34.1, and because the ICC is unknown, we assumed a moderately large value of 0.10. In turn, the design effect was calculated as 1 (34.1 − 1) × 0.1 = 4.3, resulting in an effective sample size of 1808/4.3 = 420. With unclustered data and assuming an available sample of 420 veterans to be randomized to a treatment and control group, a two-sample *t* test with 80% power and significance set at the 0.05 level would be sufficient to detect a minimum effect size (ES) of 0.19. An ES of 0.19 is conventionally considered to be a small effect.

For Aims 2 and 3, we calculated and applied a separate design effect that has been developed specifically for stepped wedge designs, and that adjusts both for clustering and for the design itself [50]. In this case, the design effect was calculated using the formula Deff = $\frac{1+\rho \Big( k tn+ bn-1}{1 + \rho \left(\frac{1}{2} ktn+ bn-1\right)}\cdot \frac{3\left(1-\rho \right)}{2 t\left( k - \frac{1}{k}\right)}$, where *ρ* is the ICC, *k* is the number of steps in the stepped wedge design, *n* is the NC of veterans per HPACT team member, *b* is the number of baseline measurement time periods, and *t* is the number of measurements after each step. The ICC is assumed again to be 0.10, with *n* estimated to be 1808/53 = 34.1 and *k*, *b*, and *t* set equal to 3, 12, and 4 in accordance with the study design. This results in a design effect of 0.17, and an effective sample size of 1808/0.17 = 10,635. Without adjusting for clustering and repeated measures, a sample of this size would be sufficient to detect an ES of 0.04, assuming 80% power and setting significance at the 0.05 level. Thus, the sample size is sufficient to detect a very small effect size in addressing Aims 2 and 3.

### Design challenges

The major design challenge in this study is contamination. However, we specifically selected GLA VA because it is a large medical center with numerous sites that generally operate separately. We believe the risk of contamination between sites, even co-located sites, is minimal because they are high service demand clinics (for example, over 100 walk-in patients per day) and complex evidence-based interventions have been difficult to implement without Facilitation in these settings.

A second design challenge is how to best get MISSION-Vet into practice within the busy HPACT programs within GLA VA. Because the HPACT providers are under pressure to serve at least 12 veterans daily, it will be important to determine if MISSION-Vet services will need to be delivered by HPACT staff or whether providers from other programs will be co-located within HPACT to deliver MISSION services. In many ways, without Facilitation, it will make the implementation as usual more of a challenge.

Finally, blinding in the traditional sense, e.g., data collectors blind to subjects’ treatment assignment, will not be possible; however, all outcome data will, in large part, be coming from secondary sources.

## Discussion

A major goal for the VA is to end veteran homelessness by 2016 (Blueprint for Excellence). One method of doing this is by improving access to and engagement in treatments for homeless veterans with co-occurring mental health and substance use disorders [51, 52]. By leveraging the intervention point of HPACT, the MISSION-Vet intervention has the potential to help accomplish this goal. Integrating MISSION into HPACT will promote access to appropriate treatments, housing stability, and recovery in this complex population with both medical and addiction care needs. However, in order for MISSION-Vet to be successful within HPACT, it is crucial that we utilize the appropriate implementation strategy.

In this case, Facilitation will be employed and has the promise to help get MISSION-Vet into practice. Because Facilitation has been used extensively as an implementation strategy in VA [19, 53], we know that it has worked well in similar settings. However, our study design will allow us to thoroughly evaluate how useful it is in the case of HPACT and MISSION-Vet. A strength of this study is the flexibility that a basic stepped wedge design offers; it is not uncommon to modify aspects of the implementation within a stepped wedge design as you turn on sites and learn from them, particularly in the context of health services studies where the study environment is highly dynamic [54–56]. Another advantage to using this modified stepped wedge design is that all sites over time receive the Facilitation strategy, which can be important for policy or ethical reasons [57, 58].

While the homeless veterans with co-occurring mental health problems and SUD are difficult to reach, this intervention has the potential to assist a complex population of homeless veterans in accessing and engaging in services in GLA for co-occurring mental health and substance use disorders. Reaching this population with MISSION has could promote housing stability and reduce the use of high-cost health care services like detox ER visits and acute hospitalizations due to relapse. The lessons learned in this study will be vital to nationwide implementation of MISSION-Vet within HPACT, further expanding the positive impact of this intervention.

## Abbreviations

CDW: Corporate data warehouse
COD: Co-occurring mental health and substance use disorders
CTI: Critical Time Intervention
Deff: Design effect
DRT: Dual recovery therapy
EF: External facilitator
ES: Effect size
FE: Formative evaluation
GLA: Greater Los Angeles VA
HPACT: in Homeless Patient Alignment Care Team
ICC: Intraclass correlation coefficient
IF: Internal facilitator
MISSION-Vet: Maintaining Independence and Sobriety through Systems Integration, Outreach, and Networking-Veterans Edition
SE: Summative evaluation
SUD: Substance use disorder

## References

[1] O’Toole TP, Conde‐Martel A, Gibbon JL, Hanusa BH, Fine MJ (2003). Health care of homeless veterans. J Gen Intern Med.

[2] Smith JL, Williams JW, Owen RR, Rubenstein LV, Chaney E (2008). Developing a national dissemination plan for collaborative care for depression: QUERI Series. Implement Sci.

[3] O’Toole TP, Johnson EE, Aiello R, Kane V, Pape L (2016). Tailoring care to vulnerable populations by incorporating social determinants of health: the Veterans Health Administration’s “Homeless Patient Aligned Care Team” Program. Prev Chronic Dis.

[4] O’Toole TP, Bourgault C, Johnson EE, Redihan SG, Borgia M, Aiello R, Kane V (2013). New to care: demands on a health system when homeless veterans are enrolled in a medical home model. Am J Public Health.

[5] O’Toole TP (2010). Primary care at the Providence VA Medical Center: challenges, opportunities and innovations. Med Health Rhode Island.

[6] Smelson D, Kalman D, Losonczy MF, Kline A, Sambamoorthi U, Hill LS, Castles-Fonseca K, Ziedonis D (2012). A brief treatment engagement intervention for individuals with co-occurring mental illness and substance use disorders: results of a randomized clinical trial. Community Ment Health J.

[7] Smelson D, Losonczy M, Castles-Fonseca K, Stewart P, Kaune M, Ziedonis D (2005). Preliminary outcomes from a booster case management program for individuals with a co-occuring substance abuse and a persistent psychiatric disorder. J Dual Diagn.

[8] Smelson D, Losonczy M, Castles-Fonseca K, Sussner B, Rodrigues S, Kaune M, Ziedonis D (2005). Preliminary outcomes from a community linkage intervention for individuals with co-occurring substance abuse and serious mental illness. J Dual Diagn.

[9] Smelson D, Losonczy M, Ziedonis D, Castles-Fonseca K, Kaune M (2007). Six month outcomes from a booster case management program for individuals with a cooccurring substance abuse and a persistent psychiatric disorder. Eur J Psychiatry Clin Neurosci.

[10] Smelson D, Kalman D, Losonczy MF, Kline A, Sambamoorthi U, St Hill L, Castles-Fonseca K, Ziedonis D (2012). A brief treatment engagement intervention for individuals with co-occurring mental illness and substance use disorders: results of a randomized clinical trial. Community Ment Health J.

[11] Smelson DA, Kline A, Kuhn J, Rodrigues S, O’Connor K, Fisher W, Kane V (2013). A wraparound treatment engagement intervention for homeless veterans with co-occurring disorders. Psychiatr Serv.

[12] Cividin TM, Ottoson JM (1997). Linking reasons for continuing professional education participation with post-program application. J Contin Educ Health Prof.

[13] Ottoson JM (1997). After the applause: exploring multiple influences on application following an adult education program. Adult Educ Q.

[14] Ennett S, Ringwalt C, Thorne J, Rohrbach L, Vincus A, Simons-Rudolph A, Jones S (2003). A comparison of current practice in school-based substance use prevention programs with meta-analysis findings. Prev Sci.

[15] Kirchner JE, Ritchie MJ, Pitcock JA, Parker LE, Curran GM, Fortney JC (2014). Outcomes of a partnered facilitation strategy to implement primary care-mental health. J Gen Intern Med.

[16] Kirchner J, Ritchie M, Dollar K, Gundlach P, Smith J (2014). Implementation facilitation training manual: using external and internal facilitation to improve care in the Veterans Health Administration.

[17] Curran GM, et al. Effectiveness-Implementation Hybrid Designs: Combining Elements of Clinical Effectiveness and Implementation Research to Enhance Public Health Impact. Med Care. 2012;50(3):217–26. 10.1097/MLR.0b013e3182408812 PMC3731143 22310560

[18] Curran GM, Bauer M, Mittman B, Pyne JM, Stetler C (2012). Effectiveness-implementation hybrid designs: combining elements of clinical effectiveness and implementation research to enhance public health impact. Med Care.

[19] Bauer MS, Miller C, Kim B, Lew R, Weaver K, Coldwell C, Henderson K, Holmes S, Seibert MN, Stolzmann K, Elwy AR, Kirchner J (2016). Partnering with health system operations leadership to develop a controlled implementation trial. Implement Sci.

[20] Harvey, G. and A. Kitson, Implementing evidence-based practice in healthcare: a facilitation guide. New York: Routledge; 2015.

[21] Susser E, Valencia E, Conover S, Felix A, Tsai W-Y, Wyatt RJ (1997). Preventing recurrent homelessness among mentally ill men: a “ critical time” intervention after discharge from a shelter. Am J Public Health.

[22] Ziedonis DM, Stern R (2001). Dual recovery therapy for schizophrenia and substance abuse. Psychiatr Ann.

[23] Ziedonis D, Brady K (1997). Dual diagnosis in primary care: detecting and treating both the addiction and mental illness. Med Clin N Am.

[24] Ziedonis DM, Smelson D, Rosenthal RN, Batki SL, Green AI, Henry RJ, Montoya I, PARKS J, Weiss RD (2005). Improving the care of individuals with schizophrenia and substance use disorders: consensus recommendations. J Psychiatr Pract.

[25] Millner W, Rollnick S (2002). Motivational interviewing: preparing people for change.

[26] Marlatt G, Gordon J (1985). Relapse prevention: maintenance strategies in addictive behaviour change.

[27] Bond GR, McHugo GJ, Becker DR, Rapp CA, Whitley R (2008). Fidelity of supported employment: lessons learned from the national evidence-based practice project. Psychiatr Rehabil J.

[28] Najavits LM (2012). Expanding the boundaries of PTSD treatment. JAMA.

[29] Smelson DA, Ziedonis D, Williams J, Losonczy MF, Williams J, Steinberg ML, Kaune M (2006). The efficacy of olanzapine for decreasing cue-elicited craving in individuals with schizophrenia and cocaine dependence: a preliminary report. J Clin Psychopharmacol.

[30] Smelson DA, Sawh L, Kane V, Kuhn J, Ziedonis DM (2011). MISSION-VET Treatment Manual.

[31] Smelson DA, Sawh L, Rodrigues S, Munoz EC, Marzilli A, Tripp J, Ziedonis DM (2011). The MISSION-VET Consumer Workbook.

[32] Smelson DA, Zaykowski H, Guevermont N, Siegfriedt J, Sawh L, Modzelewski D, Tsemberis S, Kane V (2016). Integrating permanent supportive housing and co-occurring disorders treatment for individuals who are homeless. J Dual Diagn.

[33] Ajzen IFM (1977). Attitude-behavior relations: a theoretical analysis and review of empirical research. Psychol Bull.

[34] Rosenheck RA (2001). Organizational process: a missing link between research and practice. Psychiatr Serv.

[35] Stetler CB, Legro MW, Rycroft-Malone J, Bowman C, Curran G, Guihan M, Hagedorn H, Pineros S, Wallace CM (2006). Role of “external facilitation” in implementation of research findings: a qualitative evaluation of facilitation experiences in the Veterans Health Administration. Implement Sci.

[36] Stetler CB, McQueen L, Demakis J, Mittman BS (2008). An organizational framework and strategic implementation for system-level change to enhance research-based practice: QUERI Series. Implement Sci.

[37] Owen RR, Drummond KL, Viverito KM, Marchant K, Pope SK, Smith JL, Landes RD (2013). Monitoring and managing metabolic effects of antipsychotics: a cluster randomized trial of an intervention combining evidence-based quality improvement and external facilitation. Implement Sci.

[38] Hagedorn H, Hogan M, Smith JL, Bowman C, Curran GM, Espadas D, Kimmel B, Kochevar L, Legro MW, Sales AE (2006). Lessons learned about implementing research evidence into clinical practice. J Gen Intern Med.

[39] Association D-AP (2013). Diagnostic and statistical manual of mental disorders.

[40] World Health Organization. The ICD-10 classification of mental and behavioural disorders: clinical descriptions and diagnostic guidelines. Geneva: World Health Organization; 1992.

[41] Hussey MA, Hughes JP (2007). Design and analysis of stepped wedge cluster randomized trials. Contemp Clin Trials.

[42] Gray MJ, Elhai JD, Schmidt LO (2007). Trauma professionals’ attitudes toward and utilization of evidence-based practices. Behav Modif.

[43] Jameson JP, Chambless DL, Blank MB (2009). Empirically supported treatments in rural community mental health centers: a preliminary report on current utilization and attitudes toward adoption. Community Ment Health J.

[44] Walrath CM, Sheehan AK, Holden EW, Hernandez M, Blau G (2006). Evidence-based treatments in the field: a brief report on provider knowledge, implementation, and practice. J Behav Health Serv Res.

[45] Aarons GA, McDonald EJ, Sheehan AK, Walrath-Greene CM (2007). Confirmatory factor analysis of the Evidence-Based Practice Attitude Scale in a geographically diverse sample of community mental health providers. Adm Policy Ment Health.

[46] Damschroder LJ, Aron DC, Keith RE, Kirsh SR, Alexander JA, Lowery JC (2009). Fostering implementation of health services research findings into practice: a consolidated framework for advancing implementation science. Implement Sci.

[47] Boyatzis, R.E., Transforming qualitative information: thematic analysis and code development. Thousand Oaks: Sage Publishing; 1998.

[48] Champely S (2009). pwr: basic functions for power analysis.

[49] RDevelopment, C., TEAM 2009: R: A language and environment for statistical computing. Vienna, Austria. 2012. Internet: http://www.R-project.org.

[50] Woertman W, de Hoop E, Moerbeek M, Zuidema SU, Gerritsen DL, Teerenstra S (2013). Stepped wedge designs could reduce the required sample size in cluster randomized trials. J Clin Epidemiol.

[51] O’Connell M, Kasprow W, Rosenheck RA (2010). National dissemination of supported housing in the VA: model adherence versus model modification. Psychiatr Rehabil J.

[52] Tsai J, Kasprow WJ, Rosenheck RA (2013). Latent homeless risk profiles of a national sample of homeless veterans and their relation to program referral and admission patterns. Am J Public Health.

[53] Smelson DA, Kline A, Hills S, Mizzeli A, Trip J (2007). The MISSION Consumer Workbook.

[54] Hemming KLR, Girling AJ (2015). Stepped-wedge cluster randomised controlled trials: a generic framework including parallel and multiple-level designs. Statist Med.

[55] Handley MA, Schillinger D, Shiboski S (2011). Quasi-experimental designs in practice-based research settings: design and implementation considerations. J Am Board Fam Med.

[56] Mdege ND, Man MS, Taylor Nee Brown CA, Torgerson DJ (2011). Systematic review of stepped wedge cluster randomized trials shows that design is particularly used to evaluate interventions during routine implementation. J Clin Epidemiol.

[57] Brown CA, Lilford RJ (2006). The stepped wedge trial design: a systematic review. BMC Med Res Methodol.

[58] King GGE, Ravishankar N, Moore RT, Lakin J, Vargas M, Tellez-Rojo M, Hernandez Avila JE, Hernandez Avila M, Hernandez Llamas H (2007). A “politically robust” experimental design for public policy evaluation, with application to the Mexican Universal Health Insurance Program. J Policy Anal Manage.

